# First-Line (1L) Treatment Decision Patterns and Survival of Hormone Receptor (HR)-Positive/HER2-Negative Advanced Breast Cancer (ABC) Patients in a Latin American (LATAM) Public Institution

**DOI:** 10.3390/curroncol31120581

**Published:** 2024-12-09

**Authors:** Guillermo Valencia, Patricia Rioja, Miguel Chirito, Olenka Peralta, Jorge Sánchez, Connie Rabanal, Raúl Mantilla, Zaida Morante, Hugo Fuentes, Carlos Castaneda, Tatiana Vidaurre, Cristian Pacheco, Silvia Neciosup, Henry L. Gomez

**Affiliations:** 1 Department of Medical Oncology, Instituto Nacional de Enfermedades Neoplásicas (INEN), Lima 15036, Peru; prioja@inen.sld.pe (P.R.); miguelchirito10@gmail.com (M.C.); olenka.peralta1195@gmail.com (O.P.); jorge.sanzal@gmail.com (J.S.); crabanal@inen.sld.pe (C.R.); zmorante@inen.sld.pe (Z.M.); hfuentes@inen.sld.pe (H.F.); ccastaneda@inen.sld.pe (C.C.); tvidaurre@inen.sld.pe (T.V.); cpacheco@inen.sld.pe (C.P.); sneciosup@inen.sld.pe (S.N.); 2 Grupo de Estudios Clínicos Oncológicos del Perú (GECOPERU), Lima 15038, Peru; henry.gomez@auna.org; 3 Faculty of Natural Sciences and Mathematics, Universidad Nacional Federico Villareal, Lima 15001, Peru; rmantilla@inen.sld.pe; 4 Faculty of Medicine, Universidad de Piura, Piura 20001, Peru; 5 Faculty of Medicine, Universidad Científica del Sur, Lima 15067, Peru; 6 Faculty of Medicine, Universidad Peruana Cayetano Heredia, Lima 12175, Peru; 7 Oncosalud—AUNA, Lima 15036, Peru

**Keywords:** advanced breast cancer, HR positive/HER2 negative, chemotherapy, endocrine therapy, overall survival, sequencing treatment, Peru

## Abstract

Advanced breast cancer is an incurable disease, with a median overall survival of 3 years, including in countries without access problems. Although chemotherapy is reserved in some cases, it is still used in many countries as a first-line therapy. The aim of our study is to evaluate the first-line treatment choices and the factors that influence therapeutic decisions. A retrospective analysis was conducted of hormone receptor (+)/HER2 (−) advanced breast cancer patients classified into three groups according to the first-line and second-line treatment received: endocrine therapy–chemotherapy, endocrine therapy–endocrine therapy and chemotherapy–endocrine therapy. Additionally, we explored the overall survival of sequencing therapy groups. First-line chemotherapy was chosen in 34% of patients. Also, around 60% of our patients met the “aggressive disease” criteria from the RIGHT Choice trial, justifying the use of chemotherapy in a population with poor prognosis. Furthermore, de novo and progressive disease were prognostic factors that influenced the use of chemotherapy as a first-line treatment. Regarding overall survival, the sequencing treatment groups in this trial saw an increase in survival compared with patients of the MONALEESA trials (endocrine therapy alone arms). No significant differences in progression-free survival or overall survival were found in the treatment sequencing groups. There was a higher use of chemotherapy as a first-line therapy, with de novo and “aggressive disease” criteria being the main factors to influence the decision.

## 1. Introduction

Breast cancer is the most frequently diagnosed cancer in women and leads in cancer-related mortality. The American Cancer Society estimates 313,510 new cases and 42,780 deaths in American patients with breast cancer in 2024, with 5% of metastatic disease at diagnosis [1]. Breast cancer is also the most frequently diagnosed tumor among women in Latin America (LATAM) and the Caribbean, with an increase both in incidence and mortality rates [2]. In Peru, GLOBOCAN 2022 showed that breast cancer ranked second in incidence (7797 new cases, both sexes) and seventh in mortality (1951 deaths) [3]. A report of the Instituto Nacional de Enfermedades Neoplásicas (INEN) in Peru reported that breast cancer ranked second place in terms of incidence (25 ’344 new cases), with 10.41% of patients in stage IV de novo between the years 2000 and 2020 [4]. Moreover, according to the Cancer Registry of Metropolitan Lima (2013–2015), breast cancer ranks first in incidence and mortality in women in the capital [5]. The last report of the Peruvian Ministry of Health in the first quarter of 2024 reports an occurrence of 28% of stage III cases and 31% of stage IV cases in the country [6]. Breast cancer is considered a serious issue in Peruvian health systems; a law has been approved to manage breast cancer as a national priority along with cervical cancer [7].

Advanced breast cancer (ABC) is an incurable disease, with a median overall survival (OS) of about 3 years (regardless of subtype), with a 5-year survival of 25% even in countries with more access to innovative oncologic drugs; this situation has not changed in decades [8]. About 60–70% of breast cancers are HR (+)/HER2 (−). Patients with HR (+)/HER2 (−) ABC with no visceral crisis are treated with endocrine therapy (ET) alone or in combination with targeted agents, and they benefit from sequential use of ET at disease progression. The optimal sequence for ET is not well defined. Treatment choice would depend on previous drugs, tolerance to therapy, patient preferences, and access to oncologic agents. According to international and local clinical practice guidelines (CPGs), the preferred 1L therapy for postmenopausal or premenopausal women receiving ovarian function suppression with an LHRH agonist HR (+)/HER2 (−) ABC patients is an aromatase inhibitor (AI) in combination with a CDK 4/6 inhibitor [9,10,11]. In addition, there is evidence of monotherapy with ET that supports its use as 1L therapy [12]. After progression, the following treatment options are recommended: fulvestrant +/− CDK 4/6 inhibitor [13,14,15], AI + CDK 4/6 inhibitor (if previously not received CDK 4/6 inhibitor), alpelisib (PI3K inhibitor) + ET for those with tumor with PIK3CA mutations [16], capivasertib + fulvestrant for patients with PIK3CA- or-AKT1 activating mutations or PTEN alterations [17], elascentrant for estrogen receptor 1 (ESR1)-activating mutations [18], everolimus (mTOR inhibitor) + ET [19], AI +/− fulvestrant [20], olaparib or talazoparib for BRCA1/2 mutation carriers [21], or CT. Recently, an antibody–drug conjugate (ADC) named trastuzumab deruxtecan (T-DXd) was approved for refractory HR (+)/HER2 ABC patients who received one or two previous lines of CT [22]. Although many of these innovative therapies are available in high-income countries, most low- and middle-income countries (LMICs) do not have access to most of them.

Chemotherapy is reserved for hormone refractory HR (+)/HER2 (−) patients, symptomatic visceral metastases, when there is rapid clinical progression with a need for rapid disease control, and in cases of visceral crisis (in metastatic breast cancer, this is defined as severe organ dysfunction, which involves severe signs/symptoms, and rapid progression of disease that can be assessed with laboratory studies) [23]. Combination CT usually provides a higher objective response rate (ORR) and longer time to progression, where a rapid response is needed [24]; however, it is associated with an increase in toxicity and little survival benefit [25,26]. Sequential CT is preferred over combination since it produces fewer adverse events, and both have comparable benefits in overall survival (OS) [27,28]. However, CT is not currently tailored to molecular profiles, except for platinum for BRCA1/2 mutation carriers [29].

To justify CT use, we used the “aggressive disease” criteria from the RIGHT Choice trial [30], a phase II trial which evaluates CDKi 4/6 + ET vs. CT in peri/premenopausal patients with HR (+)/HER2 (−) patients (*n* = 222) as a 1L treatment. Patients must meet at least one of the following criteria for combination CT to be considered necessary: (a) symptomatic visceral metastases, (b) rapid disease progression or impending visceral involvement, and (c) markedly symptomatic non-visceral disease if the treating physician chooses to administer CT to rapidly palliate the patient’s symptoms. Patients in this study did receive CDKi 4/6 since it was not approved for use in public institutions until April 2024.

We explore 1L treatment patterns (CT vs. ET) with HR (+) ABC Peruvian patients and potential prognostic factors that could influence treatment decisions (Peru is a middle-income country). Also, we analyze the impact of survival in sequencing treatment groups.

## 2. Materials and Methods

The study population corresponded to Peruvian patients aged ≥18 years with histologically confirmed stage IV (de novo, unresectable locally advanced/recurrent) HR-positive/HER2 (−) breast cancer treated in one public oncological institution (INEN) (*n* = 143) during March 2015–August 2023. Peri/premenopausal women received ovarian blockade.

A retrospective analysis of medical case data was performed. Patients were classified into three groups according to the treatment received in the 1L and 2L, ET-CT, ET-ET, and CT-ET, between March 2015 and August 2023 in one public oncological institution (INEN). We evaluated the patterns of 1L treatment decision patterns and the factors that influence or are associated with the choice of 1L treatment (CT vs. ET) and its outcomes.

Differences between the types of 1L treatment (ET and CT) were assessed for quantitative variables with the *t*-test or the non-parametric Mann–Whitney test, after verification of the assumption of normal distribution of the quantitative variable.

The association of qualitative variables and the 1L treatment was evaluated with the Chi-square test using contingency tables, verifying the condition of having at least 20% of the cells with expected values less than 5; if the condition was not met, the categories of the qualitative variable involved were grouped. In the 2 × 2 contingency tables, the Yates correction was applied. Univariate and multivariate logistic regression models were fitted, with variables showing significant differences or associations with the type of 1L treatment analyzed to assess their effect, by odds ratio (OR), on the probability of receiving CT as 1L.

Efficacy was measured in terms of progression-free-survival (PFS) and overall survival (OS). Estimates of OS and PFS were carried out with the Kaplan–Meier method to generate survival curves, and differences in survival according to characteristics of interest were evaluated with the log rank test. The Cox regression model was used to evaluate the association between clinical pathologic variables with PFS and OS. A *p* < 0.05 value (SPSS) was considered as a significant difference. The Software R version 4.3.2 was used for calculations and graphs.

Follow-up information was obtained from patient files. Additionally, survival status was verified from the Peruvian government web page (RENIEC) if there was not any clearly documented follow-up.

## 3. Results

### 3.1. Patients

Table 1 shows clinical pathological features. A total of 143 female patients with HR (+)/HER2 (−) ABC were included. The median age was 54 years old (range 24–85). Most patients had a good performance status (ECOG 0–1: 90%). Regarding menopausal status, 64% were postmenopausal; 29% of patients were metastatic de novo, and 69% were the luminal B subtype [defined as a subtype that typically has estrogen receptor (ER) proteins but not the progesterone receptor (PgR) inside them; patients also often have high levels of the ki67 protein (greater than 20%). Luminal B tends to grow more quickly, with a higher grade and poorer prognosis compared with the luminal A subtype] [31]. At diagnosis, 6% of patients had “visceral crisis”, and all of them received CT. Regarding 1L treatment decision, 66% of patients received ET and 34% received CT, respectively. Table 1 also shows a significantly higher proportion of patients with stage IV de novo who received 1L CT than ET (45.8% and 16.7%), while there is a trend of using 1L ET in patients with lymph node metastases (25% vs. 12%). In patients who received 1L ET, the majority used fulvestrant (52%), while patients who initiated 1L CT used anthracyclines (doxorubicin–cyclophosphamide, AC) (19%).

Table 2 shows that de novo and progressive disease have a significant effect on the possibility of receiving 1L CT, with a 4.37 (1.98–9.94, *p* < 0.001) and 3.78 (1.23–11.68, *p* = 0.01) times higher likelihood of receiving CT compared with patients with recurrent disease, respectively.

### 3.2. Survival Outcomes

#### 3.2.1. Progression-Free Survival

**Figure 1:**
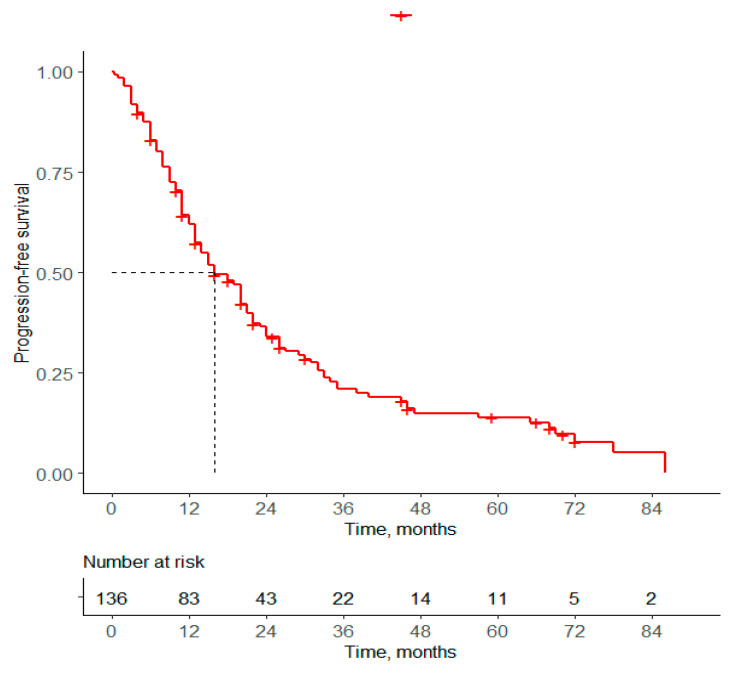
Median 1L PFS of Peruvian patients with HR (+)/HER2 (−) ABC.

**Figure 2:**
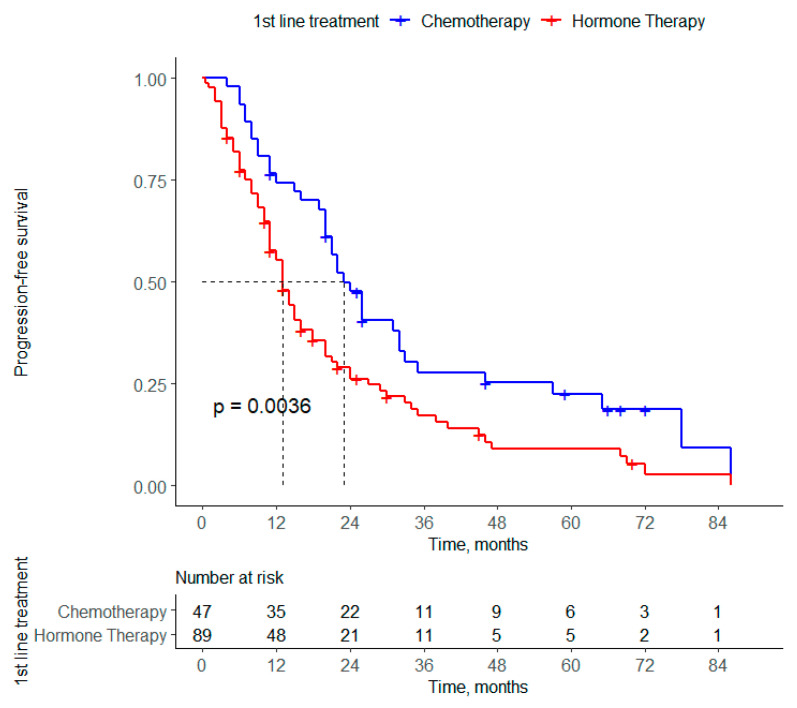
Median 1L CT vs. 1L ET of Peruvian patients with HR (+)/HER2 (−) ABC.

After a median follow-up of 15 months (1–86), the PFSs for 1L at 12, 36 and 60 months were estimated to be 61.9%, 20.8% and 13.9%, respectively. Figure 1 shows that the median PFS for 1L was estimated to be 16 months. Figure 2 shows that the median PFS for 1L is statistically longer in patients who received CT vs. ET (23 vs. 13 months, *p* = 0.0036), respectively). No significant difference was found in PFS in terms of sequential treatments according to the 1L and 2L treatment received.

#### 3.2.2. Overall Survival

**Figure 3:**
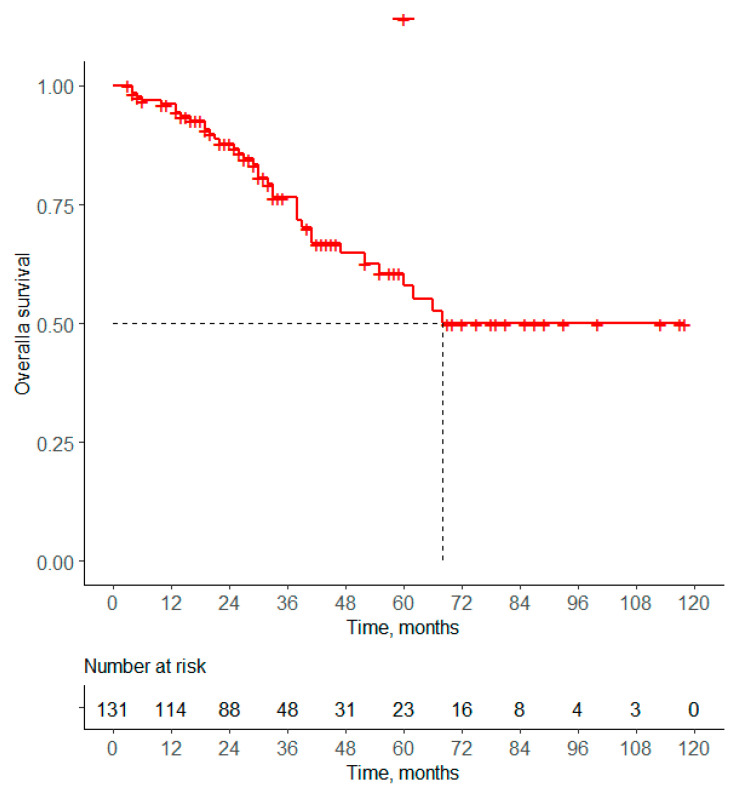
Median OS of Peruvian patients with HR (+)/HER2 (−) ABC.

The distribution of patients according to the sequence of treatments received in the 1L and 2L was as follows: 33% received ET-ET, 31% received ET-CT, and 29% received CT-ET, respectively. With a median follow-up of 29 months (range: 3–118), OS rates at 12, 36, and 60 months were estimated to be 96.1%, 76.4%, and 57.8%, respectively. Figure 3 shows that the median OS was estimated at 68 months. Table 3 shows that no significant differences were found in OS regarding the sequencing treatment of three groups.

#### 3.2.3. Patients with “Aggressive Disease”

Of the total of our patients treated with 1L CT (*n* = 51), 54% met the “aggressive disease” criteria from RIGHT Choice trial. Counting 6% of patients with visceral crisis at diagnosis, 60% of our patients met the clinical criteria of “poor prognosis” for receiving CT.

Of the total of our patients who received CT (excluding visceral crisis) and met the aggressive disease criteria, this definition includes the following: 96% of patients met the “rapid disease progression” criteria, 54% had “markedly symptomatic non-visceral metastases” (most frequently seen was multiple bone metastases with spinal cord compression requiring palliative radiotherapy, 57%), and 23% had “symptomatic visceral metastases”. Table 4 compares our patients treated with 1L CT vs. the population of the RIGHT Choice vs. MONALEESA trials’ (2, 3, and 7) criteria. Compared with the MONALEESA trials, we had more premenopausal (38% vs. 20% in MONALEESA-2 and 3) and de novo (46% vs. 35% in all MONALEESA trials) patients, while we had lower rates of visceral metastases compared with the MONALEESA and RIGHT Choice trials (46%, 60%, and 50%, respectively).

Of the total patients who received CT and did not meet the aggressive disease criteria (*n* = 22), 50% were postmenopausal without previous treatment (met criteria for MONALEESA-2), 32% were premenopausal (met criteria for MONALEESA-7), and 18% relapsed during adjuvant treatment (met criteria for MONALEESA-3).

## 4. Discussion

In the management of HR (+)/HER2 (−) ABC patients, ET + CDKi 4/6 is recommended according to international and local clinical practice guidelines (CPGs) and consensus [32,33,34]. The role of CT is reserved for visceral crisis and hormone refractory patients (as a later-line therapy option since novel endocrine agents and antibody–drug conjugates such as trastuzumab deruxtecan are now changing the landscape) [35]. This Peruvian study enrolled more patients with ECOG 2 and a higher proportion who received upfront CT than other studies reported in the literature (34% vs. 20–25%) [36]. Also, we included 29% de novo patients, similar to rates reported in pivotal trials that use CDKi 4/6, including the MONALEESA studies (34%), and LATAM countries (the incidence of de novo disease at diagnosis can reach 30–50% of cases).

Our trial demonstrated that CT is still used in a higher proportion (34%) of HR (+)/HER2 (−) ABC patients. A significant difference was found with de novo and progressive disease (defined as cancer that continues to grow, the appearance of new metastases during treatment with curative intent, or a tumor at least 20% larger than the basal size at the start of treatment) [37]; CT use is preferred over ET. These data reinforce the poor prognosis of patients with a high tumor burden or symptomatic disease who require a rapid response with systemic therapies, especially when a visceral crisis occurs [38]. For some oncologists, there is a perception that despite CT being superior to ET in terms of visceral involvement and a short disease-free interval (as it can induce higher response rates and more rapid responses), it is clearly more toxic [39,40]. Currently, this concept regarding CT has changed since CDKi 4/6 (ribociclib) has demonstrated benefits over CT in the RIGHT Choice trial in terms of PFS (21.8 vs. 12.8 months, HR 0.61, *p* = 0.003), and it has demonstrated a similar overall response rate (ORR: 66.1% vs. 66.8%, HR 0.76) in clinically aggressive premenopausal HR (+)/HER2 (−) ABC patients [41].

It is important to note that 54% of our patients who received 1L CT met the “aggressive disease” criteria of the RIGHT Choice trial, with a high occurrence (96%) of the “rapid disease progression” definition reported for the decision to use CT. Compared with the RIGHT Choice trial, our study included 60% (54% aggressive disease + 6% visceral crisis) of patients who were considered to have a “poor prognosis”; therefore, CT can be justified, especially in patients with de novo disease who are diagnosed with a high burden of disease and are symptomatic. In addition, more than half of our patients had “markedly symptomatic non-visceral metastases”, reported as multiple bone metastases with spinal cord compression that required radiotherapy (57%), showing the behavior of the disease in this Peruvian population. Despite the fact that LATAM patients face barriers in terms of access to radiation therapy (RT) units, machines and qualified personnel, Peru has made progress in radiotherapy, including in the number of machines being available [42,43]. At present, there are 28 radiotherapy machines around the country, mainly in the capital. It is important to note that all patients in the study who presented with bone metastases and spinal cord compression received palliative radiotherapy at our institution due to machine access.

Peru has a high prevalence of stage III/IV breast cancer despite implementations of a program for breast cancer screening along the country. Additionally, cultural, economic, and geographic barriers contribute to late diagnosis, with a high burden of advanced disease and inadequate access to medical resources/specialized cancer care and research [44,45].

For 40% of patients who received 1L CT and met the MONALEESA trial criteria, these patients did not receive CDKi 4/6 + ET due to access issues (no access to CDKi 4/6). We used the MONALEESA trials as comparators since they showed significant improvements in OS. In Peru, most public oncological institutions have limited access to therapies for HR (+)/HER2 (−) ABC. Regarding ET, fulvestrant has only been accessible in a public institution (INEN) since 2019 as a 1L and 2L treatment (after progression to AI); no other options except tamoxifen, anastrozole, or exemestane are available as ET in other public oncologic institutions. Moreover, the most important reason to prescribe CT in HR (+)/HER2 (−) Peruvian patients is related to a lack of access in terms of sequencing; there are no targeted therapies (CDKi) for these patients. Some progress has been made to improve access for HR (+)/HER2 (−) ABC patients in Peru. First, in 2019, fulvestrant was approved for INEN as a 1L and 2L therapy for institutional use [46]. Second, in May 2021, the INEN approved a local CPG (technical document) regarding multidisciplinary management of ABC for national reference and application in oncologic institutions of the Ministry of Health (MINSA) [47]. Also, in August 2021, a national law (Ley Nacional del Cancer) was published and declared breast cancer as a national priority for public health systems. Third, in April 2024, ribociclib + fulvestrant was approved as a 2L therapy for HR (+)/HER2 (−) ABC patients by the National Network for the Evaluation of Health Technologies (RENETSA, a local health technology evaluator) for national use [48].

In routine clinical practice, some reports have shown that a high rate of HR (+)/HER2 (−) ABC patients receive CT. A Chinese trial mentioned that less than one quarter of patients initiate 1L ET (*n* = 402) compared with those who receive CT (*n* = 1445). Patients who receive 1L ET are significantly older, with later recurrence after adjuvant therapy, with a lower rate of visceral involvement [49].

Some trials have shown that CT is still used as a 1L treatment in some countries. A study which investigated potential prognostic clinical and pathological factors for patients with luminal-like ABC and their outcomes reported that the luminal B subtype (HR 1.82), an age ≥ 70 years (HR 1.79) and >1 site of metastases (HR 2.15) had prognostic value in predicting OS. Furthermore, the presence of liver (visceral) metastases and HER2 (+) were associated with more CT use. There was no statistical difference in PFS and OS between 1L CT vs. ET [50]. Compared with our trial, we found a high proportion of patients with lymph node metastases who started 1L ET instead of visceral involvement (this term included liver, lung, and other visceral metastases) [51].

One valuable point reported in the literature that has been noted is that the median 1L PFS with CT was longer than ET (23 vs. 13 months) [52], mainly associated with the trend of its use in visceral metastases, ensuring a longer survival probably due to the time of exposure to continuous CT. This point is supported by data from a meta-analysis showing that longer 1L CT increases PFS (HR 0.64, *p* < 0.001), and OS (HR 0.91, *p* = 0.046) compared with shorter courses, but the modest increase in OS should be balanced against adverse events [26]. Longer CT regimens do not impact quality of life within clinical trials. More taxanes and capecitabine and less anthracyclines are prescribed [53]. The most common CT regimen used in our patients was AC. In the CT-ET group, ET as a “maintenance” therapy can be used after a response to CT [54]. Regarding ET-CT (31% of patients), its use is not rare; a retrospective trial found that 40% received 1L ET before CT, despite CPGs recommending ET as a 2L or subsequent therapy [55].

Regarding OS, the rate of our patients (5-year OS rate: 57.8%) was numerically longer than that reported in the MONALEESA-2 trial (43.9% in ET alone group), MONALEESA-7 trial (4-year OS rate 50% in ET alone group), and MONALEESA-3 trial (42.1% in ET alone group).

Our results have potential limitations. First, the analysis is retrospective, based on medical records. Second, the sample size is small and could affect the survival outcomes. Third, this study is a real-world-data (RWD) report, which generally has different results than pivotal trials (the latter include highly selective populations).

High-quality international and local guidelines have established a clear sequenced treatment for HR (+)/ABC (−) patients [56]. However, in countries with limited resources, local consensus [57] and adapted guidelines [58] provide recommendations of systemic treatment of ABC for clinicians, public health leaders, patients and policy makers to support the use of available therapies and justifying CT use. In 1L treatment for HR (+)/HER2 (−) ABC patients, when an aromatase inhibitor + CDKi 4/6 is unavailable, use ET alone. For life-threatening disease, use single-agent CT. In 2L treatment, the recommendation depends on prior therapy; for cases of limited access, use tamoxifen or CT. These adapted recommendations arise from the need for treatment sequencing mainly due to the lack of access to more effective high-cost oncologic therapies for HR (+)/HER2 (−) ABC in most LATAM countries, whose health care systems have unique characteristics [59].

## 5. Conclusions

One-third of HR (+)/HER2 (−) ABC Peruvian patients received 1L CT. The presence of de novo and progressive disease were prognostic factors in Peruvian patients for choosing 1L CT, whereas there is a trend that lymph node metastases were associated with the choice of ET. Moreover, around 60% of our patients who received 1L CT met the “aggressive disease” criteria, with “rapid disease progression” (96%) the most frequent definition presented in our patients who received 1L CT, followed by “markedly symptomatic non-visceral metastases” (54%) (the most common being multiple bone metastases, which cause spinal cord compression and require palliative radiotherapy). These findings are explained by the high burden of advanced disease and behavior at diagnosis in our population. Additionally, patients who received 1L CT and met the RIGHT Choice criteria had more de novo disease and visceral metastases than those who met the MONALEESA-2, 3, and 7 trial criteria. In contrast, 40% of our 1L CT patients did not meet the aggressive disease criteria; therefore, they did not receive CDKi 4/6 due to the lack of access in our public institutions.

Treatment sequencing (ET-ET, ET-CT, CT-ET) in HR (+)/HER2 (−) ABC Peruvian patients numerically increases 1L PFS (especially 1L CT vs. ET) and OS rates compared with the ET alone groups of pivotal trials which evaluated CDKi 4/6 (MONALEESA trials), mainly due to the time of exposure to continuous CT. The use of these sequences is supported by international and local consensus adapted to LMIC. There were no statistical differences in PFS or OS for the sequential treatments groups. Currently, Peruvian health systems are making efforts to improve survival outcomes, including updating local breast cancer CPGs and increasing access to more effective oncologic therapies such as CDKi 4/6 for ABC patients.

## Figures and Tables

**Table 1:** Variables under study according to first-line (1L) treatment.

		1L Treatment		
	All Patients *n* = 143	Endocrine Therapy (ET) *n* = 95	Chemotherapy (CT) *n* = 48	*p*-Value
Age at diagnosis, years				
Average/range	54.2 (24–85)	54.5 (29–85)	53.6 (24–84)	0.707
Age groups, years				
<50	51 (35.7%)	33 (34.7%)	18 (37.5%)	
≥50	92 (64.3%)	62 (65.3%)	30 (62.5%)	0.745
ECOG at diagnosis				
0–1	133 (93.0%)	89 (93.7%)	44 (91.7%)	
2	10 (7.0%)	6 (6.3%)	4 (8.3%)	0.921
De novo disease				
Yes	41 (28.7%)	19 (20.0%)	22 (45.8%)	
No	102 (71.3%)	76 (80.0%)	26 (54.2%)	**<0.001**
Metastatic sites				
Bone	85 (59.4%)	52 (54.7%)	33 (68.8%)	0.107
Lung	39 (27.3%)	22 (23.2%)	18 (37.5%)	0.071
Lymph node	29 (20.3%)	24 (25.3%)	6 (12.5%)	0.077
Locoregional	15 (10.5%)	13 (13.6%)	3 (6.3%)	0.183
Liver	13 (9.1%)	11 (11.6%)	3 (6.3%)	0.475
Contralateral	5 (3.5%)	3 (3.2%)	2 (4.2%)	1
Dermis	4 (2.8%)	2 (2.1%)	2 (4.2%)	0.866
Soft tissue	3 (2.1%)	2 (2.1%)	1 (2.1%)	1
Peritoneal	2 (1.4%)	1 (1.1%)	1 (2.1%)	1
Mediastinum	2 (1.4%)	1 (1.1%)	0 (0.0%)	1
Cutaneous	2 (1.4%)	2 (2.1%)	0 (0.0%)	0.796
Suprarenal	1 (1.4%)	1 (1.1%)	1 (2.1%)	1
Ovary	1 (0.7%)	0 (0.0%)	1 (2.1%)	0.727
Pancreas	1 (0.7%)	0 (0.0%)	1 (2.1%)	0.727
Number of metastases				
1	99 (69.2%)	66 (69.5%)	33 (68.8%)	
2	32 (22.4%)	23 (24.2%)	9 (18.8%)	
3	9 (6.3%)	5 (5.3%)	4 (8.3%)	
≥4	3 (2.1%)	1 (1.1%)	2 (4.2%)	NE
Luminal subtype				
A	40 (31.0%)	24 (28.6%)	16 (35.6%)	
B	89 (69.0%)	60 (71.4%)	29 (64.4%)	0.414
NR	14	11	3	
Intensity of HER2 expression				
0	81 (60.4%)	56 (62.2%)	25 (56.8%)	
1+/2+	53 (39.6%)	34 (37.8%)	19 (43.2%)	0.548
NR	9	5	4	
Ki-67				
<20%	27 (20.9%)	17 (20.2%)	10 (22.2%)	
≥20%	63 (48.8%)	32 (45.2%)	25 (55.6%)	0.339
NR	14	11	3	

NE: not evaluable, NR: not registered. Bold in the *p*-value column means that the result is statistically significant.

**Table 2:** Evaluation of the effect of variables to estimate the possibility of selecting 1L CT.

		Univariate	
	OR	95% CI	*p*-Value
Clinical stage IV			
Recurrent	Ref.		
Progressive	3.78	1.23–11.68	**0.019**
De novo	4.37	1.98–9.94	**<0.001**

OR: odds ratio, CI: confidence interval, Ref.: reference. Bold in the *p*-value column means that the result is statistically significant.

**Table 3:** Estimates of overall survival (OS) according to 1L treatment and sequencing.

		OS (months)		
	12	36	60	*p*-Value
All patients	96.1%	76.4%	57.8%	-
1L treatment				
Endocrine therapy (ET)	94.0%	82.4%	66.2%	
Chemotherapy (CT)	100%	71.2%	52.2%	0.36
Treatment sequencing				
ET-ET	100%	83.9%	65.4%	
ET-CT	88.0%	80.3%	64.3%	
CT-ET	100%	65.4%	52.5%	
CT-CT	100%	87.5%	58.3%	0.42

**Table 4:** Peruvian patients who received chemotherapy (CT) as first-line (1L) vs. MONALEESA trial vs. RIGHT Choice trial.

Patients (pts) Received 1L CT		Peruvian pts	MONALEESA (ML) Trials	RIGHT Choice Trial
Median age		52 (24–80) year	55 year	Premenopausal
Postmenopausal		62%	80%	0%
Premenopausal		38%	20% ML-2 20% ML-3 100% ML-7	100%
De novo (diagnosis)		46%	35%	NR
ECOG 0–1		92%	99%	99%
Luminal B subtype		66%	NR	NR
Visceral metastases		46%	60%	50%
Visceral crisis		6%	0%	50%
Met aggressive disease (RIGHT Choice) definition		54%	ND	100%
“Aggressive disease” criteria	Symptomatic visceral metastases	23%	ND	67.6%
	Rapid disease progression	96%	ND	18.5%
	Markedly symptomatic non-visceral metastases	54%	ND	14%

ML: MONALEESA, NR: Not reported, pts: patients.

## Data Availability

The data presented in this study are available upon request to the corresponding author because prior authorization is required from the institution where the patients were treated in order to maintain and respect the confidentiality and privacy of this information.

## References

[1] Siegel R., Giaquinto A.N., Jemal A. (2024). Cancer statistics, 2024. CA Cancer J. Clin..

[2] Piñeros M., Laversanne M., Barrios E., Cancela M.C., de Vries E., Pardo C., Bray F. (2022). An updated profile of the cancer burden, patterns and trends in Latin America and the Caribbean. Lancet Reg. Health Am..

[3] Sung H., Ferlay J., Siegel R.L., Laversanne M., Soerjomataram I., Jemal A., Bray F. (2021). Global cancer statistics 2022: GLOBOCAN estimates of incidence and mortality worldwide for 36 cancers in 185 countries. CA Cancer J Clin..

[4] Cancer News Cases 2000–2020 Cancer Epidemiology Unit—Instituto Nacional de Enfermedades Neoplásicas (INEN). https://portal.inen.sld.pe/.

[5] Payet E. (2021). Registro de Cáncer de Lima Metropolitana: Incidencia y Mortalidad 2013–2015.

[6] Sala Situacional de Cáncer en el Perú (2024). I Trimestre 2024. UT ENT CDC Perú. Vigilancia Epidemiológica del Cáncer Ministerio de Salud.

[7] Ley N° 31336 (2021). Ley Nacional del Cáncer. Diario Nacional El Peruano. https://busquedas.elperuano.pe/dispositivo/NL/1980284-2.

[8] Cardoso F., Spence D., Merts S., Corneliussen-James D., Sabelko K., Gralow J., Cardoso M.J., Peccatori F., Paonessa D., Benares A. (2018). Global analysis of advanced/metastatic breast cancer: Decade report (2005–2015). Breast.

[9] Slamon D.J., Diéras V., Rugo H.S., Harbeck N., Im S.A., Gelmon K.A., Lipatov O.N., Walshe J.M., Martin M., Chavez-MacGregor M. (2024). Overall survival with palbociclib plus letrozole in advanced breast cancer. J. Clin. Oncol..

[10] Hortobagyi G.N., Stemmer S.M., Burris H.A., Yap Y.S., Sonke G.S., Hart L., Campone M., Petrakova K., Winer E.P., Janni W. (2022). Overall survival with ribociclib plus letrozole in advanced breast cancer. N. Engl. J. Med..

[11] Goetz M.P., Toi M., Huober J., Sohn J.H., Trédan O., Park I., Campone M., Chen S.C., Manso L.M., Paluch-Shimon S. (2024). MONARCH 3: Final overall survival results of abemaciclib plus a nonsteroidal aromatase inhibitor as first-line therapy for HR+, HER2- advanced breast cancer. Abstract GS01-12. Cancer Res..

[12] Robertson J.F.R., Bondarenko I.M., Trishkina E., Dvorkin M., Panasci L., Manikhas A., Shparyk Y., Cardona-Huerta S., Cheung K.L., Philco-Salas M.J. (2016). Fulvestrant 500 mg versus anastrozole 1 mg for hormone receptor-positive advanced breast cancer (FALCON): An international, randomised, double-blind, phase 3 trial. Lancet.

[13] Cristofanilli M., Rugo H.S., Im S.A., Slamon D.J., Harbeck N., Bondarenko I., Masuda N., Colleoni M., DeMichele A., Loi S. (2022). Overall survival with palbociclib and fulvestrant in women with HR+/HER2- ABC: Updated exploratory analyses of PALOMA-3, a double-blind, phase III randomized study. Clin. Cancer Res..

[14] Neven P., Fasching P.A., Chia S., Jerusalem G., De Laurentiis M., Im S.A., Petrakova K., Bianchi G.V., Martin M., Nusch A. (2023). Updated overall survival from the MONALEESA-3 trial in postmenopausal women with HR+/HER2− advanced breast cancer receiving first-line ribociclib plus fulvestrant. Breast Cancer Res..

[15] Llombart-Cussac A., Sledge G., Toi M., Neven P., Sohn J.H., Inoue K., Pivot X., Okera M., Masuda N., Kaufman P.A. Final overall survival analysis of MONARCH 2: A phase 3 trial of abemaciclib plus fulvestrant in patients with hormone receptor-positive HER2-negative advanced breast cancer. Poster presentation. Proceedings of the San Antonio Breast Cancer Conference.

[16] André F., Ciruelos E.M., Juric D., Loibl S., Campone M., Mayer I.A., Rubovszky G., Yamashita T., Kaufman B., Lu Y.S. Overall survival results from SOLAR-1, a phase III study of alpelisib + fulvestrant for hormone receptor-positive, human epidermal growth factor receptor 2-negative advanced breast cancer. Proceedings of the ESMO Virtual Congress.

[17] Turner N.C., Oliveira M., Howell S.J., Dalenc F., Cortes J., Gomez Moreno H.L., Jhaveri K., Krivorotko P., Loibl S., Morales Murillo S. (2023). Capivasertib in Hormone Receptor–Positive Advanced Breast Cancer. N. Engl. J. Med..

[18] Bidard F.C., Kaklamani V.G., Neven P., Streich G., Montero A.J., Forget F., Mouret-Reynier M.A., Sohn J.H., Taylor D., Harnden K.K. (2022). Elacestrant (oral selective estrogen receptor degrader) Versus Standard Endocrine Therapy for Estrogen Receptor–Positive, Human Epidermal Growth Factor Receptor 2–Negative Advanced Breast Cancer: Results From the Randomized Phase III EMERALD Trial. Clin. Oncol..

[19] Baselga J., Campone M., Piccart M., Burris H.A., Rugo H.S., Sahmoud T., Noguchi S., Gnant M., Pritchard K.I., Lebrun F. (2012). Everolimus in postmenopausal hormone-receptor–positive advanced breast cancer. N. Engl. J. Med..

[20] Chia S., Gradishar W., Mauriac L., Bines J., Amant F., Federico M., Fein L., Romieu G., Buzdar A., Robertson J.F.R. (2008). Double-blind, randomized placebo controlled trial of fulvestrant compared with exemestane after prior nonsteroidal aromatase inhibitor therapy in postmenopausal women with hormone receptor-positive, advanced breast cancer: Results from EFECT. J. Clin. Oncol..

[21] Burstein H.J., Somerfield M.R., Barton D.L., Dorris A., Fallowfield J., Jain D., Johnston S.R.D., Korde L.A., Litton J.K., Macrae E.R. (2021). Endocrine Treatment and Targeted Therapy for Hormone Receptor–Positive, Human Epidermal Growth Factor Receptor 2–Negative Metastatic Breast Cancer: ASCO Guideline Update. J. Clin. Oncol..

[22] Modi S., Jacot W., Yamashita T., Sohn J., Vidal M., Tokunaga E., Tsurutani J., Ueno N.T., Prat A., Chae Y.S. (2022). Trastuzumab deruxtecan in previously treated HER2-low advanced breast cancer. N. Eng. J. Med..

[23] Cardoso F., Paluch-Shimon S., Senkus E., Curigliano G., Aapro M.S., André F., Barrios C.H., Bergh J., Bhattacharyya G.S., Biganzoli L. (2020). 5th ESO-ESMO international consensus guidelines for advanced breast cancer (ABC 5). Ann. Oncol..

[24] Cardoso F., Senkus E., Costa A., Papadopoulos E., Aapro M., André F., Harbeck N., Aguilar Lopez B., Barrios C.H., Bergh J. (2018). 4th ESO-ESMO International Consensus Guidelines for Advanced Breast Cancer (ABC 4). Ann. Oncol..

[25] O’Shaughnessy J., Miles D., Vukelja S., Moiseyenko V., Ayoub J.P., Cervantes G., Fumoleau P., Jones S., Lui W.Y., Mauriac L. (2002). Superior survival with capecitabine plus docetaxel combination therapy in anthracycline pretreated patients with advanced breast cancer: Phase III trial results. J. Clin. Oncol..

[26] Gennari A., Stockler M., Puntoni M., Sormani M., Nanni O., Amadori D., Wilcken N., D’Amico M., DeCensi A., Bruzzi P. (2011). Duration of chemotherapy for metastatic breast cancer: A systematic review and meta-analysis of randomized clinical trials. J. Clin. Oncol..

[27] Carrick S., Parker S., Thornton C.E., Ghersi D., Simes J., Wilcken N. (2005). Single agent versus combination chemotherapy for metastatic breast cancer. Cochrane Database Syst. Rev..

[28] Albain K.S., Calderillo-Ruiz G., Jordaan J.P., Llombart A., Pluzanska A., Pawlicki M., Melemed A.S., O’Shaughnessy J., Reyes J.M. (2004). Global phase III study of gemcitabine plus paclitaxel (GT) vs. paclitaxel (T) as frontline therapy for metastatic breast cancer (MBC): First report of overall survival. J. Clin. Oncol..

[29] Cardoso F., Colleoni M., Di Leo A., Francia G., Gennari A., Gligorov J., Llombart A. (2016). Oral chemotherapy in advanced breast cancer: Expert perspectives on its role in clinical practice. Cancer Treat. Commun..

[30] Lu Y.S., Mahidin E.I.B.M., Azim H., Eralp Y., Yap Y.S., Im S.A., Rihani J., Bowles J., Alfaro T.D., Wu J. (2023). Primary results from the randomized Phase II RIGHT Choice trial of premenopausal patients with aggressive HR+/HER2− advanced breast cancer treated with ribociclib + endocrine therapy vs. physician’s choice combination chemotherapy. Abstract GS1-10. Cancer Res..

[31] Orrantia-Borunda E., Anchondo-Nuñez P., Acuña-Aguilar L.E., Gómez-Valles F.O., Ramírez-Valdespino C.A., Mayrovitz H.N. (2022). Subtypes of breast cancer. Breast Cancer.

[32] National Comprehensive Cancer Network (NCCN) (2024). Clinical Practice Guidelines. Breast Cancer.

[33] Cardoso F., Paluch Shimon S., Schumacher-Wulf E., Matos L., Gelmon K., Aapro M.S., Bajpai J., Barrios C.H., Bergh J., Bergstaen-Nordstrom E. (2024). 6th and 7th International consensus guidelines for the management of advanced breast cancer (ABC guidelines 6 and 7). Breast.

[34] Gennari A., André F., Barrios C.H., Cortes J., de Azambuja E., DeMichele A., Dent R., Fenlon D., Glogorov J., Hurvitz S.A. (2021). ESMO Clinical Practice Guideline for the diagnosis, staging and treatment of patients with metastatic breast cancer. Ann. Oncol..

[35] Huppert L.A., Gumusay O., Idossa D., Rugo H.S. (2023). Systemic therapy for hormone receptor-positive/human epidermal growth factor receptor 2-negative early stage and metastatic breast cancer. CA A Cancer J. Clin..

[36] Brufsky A.M. (2015). Delaying Chemotherapy in the Treatment of Hormone Receptor–Positive, Human Epidermal Growth Factor Receptor 2–Negative Advanced Breast Cancer. Clin. Med. Insights Oncol..

[37] Ling Y., Xie Y.F., Wu H., Wang X., Ma J., Fan L., Liu G. (2023). Prognostic factors and clinical outcomes of breast cancer patients with disease progression during neoadjuvant systemic therapy. Breast.

[38] McAndrew N.P., Finn R.S. (2021). Clinical Review on the Management of Hormone Receptor–Positive Metastatic Breast Cancer. JCO Oncol. Pract..

[39] Barrios C.H., Sampaio C., Vinholes J., Caponero R. (2009). What is the role of chemotherapy in estrogen receptor-positive, advanced breast cancer?. Ann. Oncol..

[40] Barrios C.H. (2010). The role of chemotherapy in hormone receptor positive advanced breast cancer. Gac. Mex. Oncol. GAMO.

[41] Lu Y.S., Mahidin E.I.B.M., Azim H., Eralp Y., Yap Y.S., Im S.A., Rihani J., Gokmen E., El Bastawisy A., Karadurmus N. (2024). Final results of RIGHT Choice: Ribociclib plus endocrine therapy versus combination chemotherapy in premenopausal women with clinically aggressive hormone receptor–positive/human epidermal growth factor receptor 2–Negative Advanced Breast Cancer. J. Clin. Oncol..

[42] Pinillos L., Pinto J.A., Sarria G. (2017). History of the development of radiotherapy in Latin America. Ecancermedicalscience.

[43] Sarria G.R., Martinez D.A., Li B., Del Castillo R., Salgado A., Pinillos L., Felix A., Bobadilla I., Ferraris G., Castilho M. (2023). Radiotherapy and cancer status in Latin America: Economic analysis of investment opportunities up to 2030. Int. J. Radiat. Oncol. Biol. Phys..

[44] Araujo J.M., Gómez A.C., Zingg-De Jongh W., Ausejo J., Córdova I., Schwarz Luis J., Bretel D., Fajardo W., Saravia-Huarca L.G., Barboza-Meca J. (2023). A nationwide pilot study on breast cancer screening in Peru. Ecancermedicalscience.

[45] Ayala N., Barchuk S., Inurrigarro G., Celano C., Soriano-García J.L., Bolaños P., Mohs-Alfaro M., Tapia-González H., Perez-Martinez R., Samtani S. (2023). Status of breast cancer in Latin American: Results of the breast cancer revealed initiative. Crit. Rev. Oncol./Hematol..

[46] Valencia-Mesías G., Rioja-Viera P., Morante-Cruz Z., Toledo-Morote Y., Neciosup-Delgado S., Gómez-Moreno H. (2021). The current situation regarding the availability and accessibility of anticancer drugs for breast cancer in the Peruvian public health systems. Ecancermedicalscience.

[47] (2021). Documento Técnico: “Tratamiento Multidisciplinario del Cáncer de Mama Metastásico” Resolución Jefatural N°, 1.6.6.-2.0.2.1.-J-INEN. Instituto Nacional de Enfermedades Neoplásicas. https://portal.inen.sld.pe/guias-tecnicas/.

[48] Instituto Nacional de Salud RENETSA. https://www.gob.pe/institucion/ins/colecciones/11902-renetsa.

[49] Wu Y., Han Y., Yu P., Ouyang Q., Yan M., Wang X., Hu X., Jiang Z., Huang T., Tong Z. (2020). Endocrine therapy for hormone receptor-positive advanced breast cancer: A nation-wide multicenter epidemiological study in China. Front. Oncol..

[50] Bonotto M., Gerratana L., Arpino G., Di Maio M., De Angelis C., Iacono D., Cinausero M., Milano M., Gargiulo P., Fontanella C. (2015). First line treatment in patients with luminal-like metastatic breast cancer: A propensity score-matched analysis. Ann. Oncol..

[51] Hortobagyi G.N., Stemmer S.M., Burris H.A., Yap Y.S., Sonke G.S., Paluch-Shimon S., Campone M., Blackwell K.L., André F., Winer E.P. (2016). Ribociclib as first-line for HR-positive, advanced breast cancer. N. Engl. J. Med..

[52] Nabholtz J.M., Buzdar A., Pollak M., Harwin W., Burton G., Mangalik A., Steinberg M., Webster A., von Euler M. (2000). Anastrozole Is Superior to Tamoxifen as First-Line Therapy for Advanced Breast Cancer in Postmenopausal Women: Results of a North American Multicenter Randomized Trial. J. Clin. Oncol..

[53] Claessens A.K.M., Ibragimova K.I.E., Geurts S.M.E., Bos M.E.M.M., Erdkamp F.L.G., Tjan-Heijnen V.C.G. (2020). The role of chemotherapy in treatment of advanced breast cancer: An overview for clinical practice. Crit. Rev. Oncol. Hematol..

[54] Berruti A., Zola P., Buniva T., Bau M.G., Farris A., Sarobba M.G., Bottini A., Tampellini M., Durando A., Destefanis M. (1997). Prognostic factors in metastatic breast cancer patients obtaining objective response or disease stabilization after first-line chemotherapy with epirubicin. Evidence for a positive effect of maintenance hormonal therapy on overall survival. Anticancer. Res..

[55] Macalalad A.R., Hao Y., Lin P.L., Signorovitch J.E., Wu E.Q., Ohashi E., Zhou Z., Kelley C. (2015). Treatment patterns and duration in post-menopausal women with HR+/HER2- metastatic breast cancer in the US: A retrospective chart review in community oncology practices 2004–2010. Curr Med Res Opin..

[56] Rej R.K., Roy J., Allu S.R. (2024). Therapies for the Treatment of Advanced/Metastatic Estrogen Receptor-Positive Breast Cancer: Current Situation and Future Directions. Cancers.

[57] Valencia F., Gómez H.L., Neciosup S.P., Limón R., Torrico M., Morillas L., Torres R., Sánchez C., Araya I., Gómez R. (2024). Advanced Breast Cancer guidelines in Latin America: Assessment, adaptation, and implementation of Fifth Advanced Breast Cancer consensus guidelines. JCO Glob. Oncol..

[58] Al Sukhun S., Temin S., Barrios C.H., Antone N.Z., Guerra Y.C., Chavez-MacGregor M., Chopra R., Danso M.A., Gomez H.L., Homian N.M. (2024). Systemic treatment of patients with metastatic breast cancer: ASCO Resource-Stratified Guideline. JCO Global Oncol..

[59] Llera A.S. (2023). A fresh perspective on Latin America cancer care: Uncovering hidden messages in unconventional data sources. Lancet Reg. Health Am..

